# p70S6K as a Potential Anti-COVID-19 Target: Insights from Wet Bench and In Silico Studies

**DOI:** 10.3390/cells13211760

**Published:** 2024-10-24

**Authors:** Sharon Shechter, Rajat Kumar Pal, Fabio Trovato, Or Rozen, Matthew J. Gage, Dorit Avni

**Affiliations:** 1 Department of Chemistry, University of Massachusetts Lowell, Lowell, MA 01854, USA; sharon_shechter@student.uml.edu (S.S.); matthew_gage@uml.edu (M.J.G.); 2 Independent Free Energy Researcher, Waltham, MA 02451, USA; rajatfor2014@gmail.com; 3 Psivant Therapeutics, 451 D Street, Boston, MA 02210, USA; fabiotrovato@gmail.com; 4 Department of Natural Compound, Nutrition, and Health, MIGAL Galilee Research Institute, Kiryat Shmona 1101600, Israel; orrozen2@gmail.com

**Keywords:** cytokine release syndrome, COVID-19, SARS-CoV-2, kinases, macrophages

## Abstract

The onset of SARS-CoV-2 infection in 2019 sparked a global COVID-19 pandemic. This infection is marked by a significant rise in both viral and host kinase activity. Our primary objective was to identify a pivotal host kinase essential for COVID-19 infection and the associated phenomenon of the cytokine storm, which may lead to long-term COVID-19 complications irrespective of viral genetic variations. To achieve this, our study tracked kinase phosphorylation dynamics in RAW264.7 macrophages following SPIKE transfection over time. Among the kinases surveyed, p70S6K (RPS6KB1) exhibited a 3.5-fold increase in phosphorylation at S418. This significant change prompted the selection of p70S6K for in silico investigation, utilizing its structure bound to M2698 (PDB: 7N93). M2698, an oral dual Akt/p70S6K inhibitor with an IC_50_ of 1.1 nM, exhibited psychosis side effects in phase I clinical trials, potentially linked to its interaction with Akt2. Our secondary objective was to discover a small-molecule analogue of M2698 that exhibits a distinct binding preference for p70S6K over Akt2 through computational modeling and analysis. The in silico part of our project began with validating the prediction accuracy of the docking algorithm, followed by an OCA analysis pinpointing specific atoms on M2698 that could be modified to enhance selectivity. Subsequently, our investigation led to the identification of an analog of M2698, designated as S34, that showed a superior docking score towards p70S6K compared to Akt2. To further assess the stability of S34 in its protein–ligand (PL) complexes with p70S6K and Akt2, MD simulations were conducted. These simulations suggest that S34, on average, forms two hydrogen bond interactions with p70S6K, whereas it only forms one hydrogen bond interaction with Akt2. This difference in hydrogen bond interactions likely contributed to the observed larger root mean square deviation (RMSD) of 0.3 nm in the S34-Akt2 complex, compared to 0.1 nm in the S34-p70S6K complex. Additionally, we calculated free binding energy to predict the strength of the binding interactions of S34 to p70S6K and Akt2, which showed ~2-fold favorable binding affinity of S34 in the p70S6K binding pocket compared to that in the Akt2 binding pocket. These observations may suggest that the S34-p70S6K complex is more stable than the S34-Akt2 complex. Our work focused on identifying a host kinase target and predicting the binding affinity of a novel small molecule to accelerate the development of effective treatments. The wet bench results specifically highlight p70S6K as a compelling anti-COVID-19 target. Meanwhile, our in silico investigations address the known off-target effects associated with M2698 by identifying a close analog called S34. In conclusion, this study presents novel and intriguing findings that could potentially lead to clinical applications with further investigations.

## 1. Introduction

Coronavirus disease 2019 (COVID-19), stemming from the severe acute respiratory syndrome coronavirus 2 (SARS-CoV-2), remains a formidable infectious disease with profound implications for public health. Recent investigations have unveiled 11 novel bat coronavirus species, underscoring the extensive diversity within the coronavirus family and raising concerns about potential zoonotic transmissions [1]. Often, these newly identified species carry unseen genetic mutations, exemplified by the recently recognized BA.2.86, introducing the possibility of diverse pathological manifestations [2].

**Figure 1:**
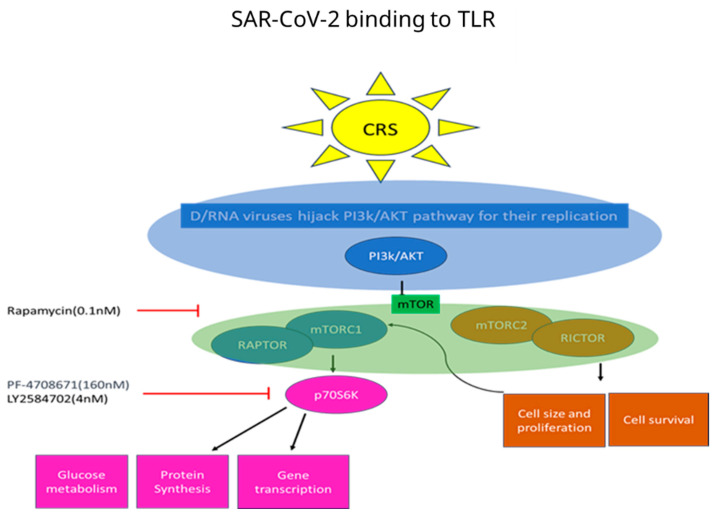
SARS-CoV-2 PI3K/mTOR binding to toll-like receptor (TLR) pathway activation. TLR recognizes viral proteins and causes phosphorylation of many kinases, including p70S6K, which can control various cellular functions. Arrows indicate activation, and blocks indicate inhibition. CRS = cytokine release syndrome. LY2584702, an ATP-competitive p70S6K, tested in phase I clinical trials as an anti-cancer agent, but trials were terminated prematurely (ClinicalTrials.gov NCT01394003). PF–4708671 is used as a specific p70S6K research tool that has not been evaluated in clinical trials.

Individuals affected by COVID-19 frequently undergo acute inflammation, also identified as cytokine release syndrome (CRS) [3], due to the robust viral replication in the epithelial cells of the upper and lower respiratory systems, leading to acute respiratory distress syndrome (ARDS) and multi-organ failure [1,4,5]. CRS is characterized by systemic immune system activation upon the virus’s entry into the body, resulting in heightened levels of pro-inflammatory cytokines and chemokines (Figure 1, [6]). Cytokines, such as tumor necrosis factor (TNF)-α [7], are proteins secreted by various immune cells (e.g., macrophages), which orchestrate the immune response by guiding these cells to sites of inflammation [8]. This process is mediated by kinases, which activate each other in a cascade of reactions [9]. Emerging evidence indicates that inhibiting pro-inflammatory signaling pathways holds promise for attenuating the inflammatory process, thereby improving clinical outcomes [10].

In the wake of the COVID-19 pandemic, substantial efforts have been directed towards identifying kinases with known drug inhibitors that are crucial for the viral life cycle and integral to COVID-19 infection and its related CRS. A wealth of data has highlighted numerous hotspots, presenting new opportunities for targeted interventions with existing drugs or novel compounds. These findings offer potential for both virus- and host-directed therapeutic strategies [11,12,13]. The kinase protein family’s pivotal role in regulating inflammatory responses induced by pro-inflammatory cytokines (e.g., interluikin-6 and TNF-α) or proteins associated with inflammatory reactions (e.g., transforming growth factor beta (TGF-β)) has been well established [14]. Moreover, kinase inhibitors demonstrated direct anti-inflammatory, cytokine-suppressing, and antifibrotic effects, underscoring their potential as anti-viral targets [9]. Consequently, an array of kinase inhibitors is currently undergoing investigation in clinical trials as potential treatments for COVID-19 [15].

The intricate relationship between host kinases and viral infections becomes apparent as these kinases govern the host’s cell cycle, innate immune signaling, and stress response in the face of viral invasion and inflammation. Furthermore, coronaviruses strategically manipulate kinase cascades to subvert host cell responses by exploiting host kinase activity for the phosphorylation of viral proteins, thereby amplifying the replication process [14]. Notably, SARS-CoV-2 has been identified as phosphorylating key phosphosites on kinases, such as cyclin-dependent kinases (CDKs), phosphoinositide 3-kinase (PI3K)/Akt, mitogen-activated protein kinases (MAPKs), ataxia telangiectasia mutated (ATM), and checkpoint kinase 1 (CHEK1), all of which play critical roles in cell cycle regulation, cell growth, motility, stress responses, and DNA damage [16]. Additionally, SARS-CoV-2 modulates various signaling pathways, including epidermal growth factor receptor (EGFR) signaling, TGF-β, and autophagy. The targeted inhibition of elevated kinases and associated signaling pathways emerges as a promising avenue for potential anti-COVID-19 drug discovery [17].

mTOR, a serine/threonine kinase, serves as the catalytic subunit in two signaling complexes designated as the mechanistic target of rapamycin complex 1 (mTORC1) and mechanistic target of rapamycin complex 2 (mTORC2, Figure 1). mTORC1 is implicated in signaling processes by phosphorylating eukaryotic initiation factor 4E-binding protein 1 (4E-BP1) and ribosomal S6 kinase 1 (S6K1). While the precise function of mTORC2 is not fully elucidated, its activity seems to be interconnected with mTORC1 through direct phosphorylation by S6K1 [18]. A key pathway operating via mTORC1 is the phosphoinositide 3 kinase (PI3K)/Akt signal transduction pathway, integral in cellular survival and proliferation [18]. Akt directly phosphorylates mTOR, leading to the activation of mTORC1, resulting in the phosphorylation of specific substrates, including S6K1 (p70S6K). Through its action on p70S6K, mTOR facilitates processes such as ribosome biogenesis and translation elongation [18]. p70S6K, situated downstream of mTOR, represents an isoform of S6K tasked with regulating cell size by influencing mRNA transcription and splicing, protein synthesis, cell apoptosis, and metabolism [19]. This kinase is regulated through multiple phosphorylation events within its catalytic, linker, and pseudo-substrate (autoinhibitory) domains, subsequently leading to the specific phosphorylation of ribosomal protein S6.

In their 2023 study, Fritch and colleagues investigated phosphorylation changes induced by SARS-CoV-2 infection, revealing activation of several kinase pathways, such as MAPK, mTOR, PI3K/Akt, ERBB, and ephrin receptor signaling. This study highlights the complex modulation of cellular signaling in response to viral infection [20]. Additionally, recent work has shown that the SARS-CoV-2 virus exploits the mTOR-signaling pathway to progress its infection [21,22,23]. These findings align with previous studies demonstrating that various DNA/RNA viruses exploit the PI3K/Akt/mTOR pathway to facilitate replication within mammalian host cells [24]. Moreover, the mTOR signaling pathway can trigger autophagy and suppress viral protein synthesis [25]. Treatment with PI3K/mTOR inhibitors effectively suppressed coronavirus replication at nanomolar concentrations. Moreover, PI3K is recognized for its role in regulating diverse inflammatory responses [26].

Figure 1 illustrates the immune cascade activated in response to viral recognition involving p70S6K, among other components. Specifically, toll-like receptors (TLRs), which are innate immune receptors, recognize viral proteins and initiate the production of pro-inflammatory cytokines to combat infection. Upon activation, PI3K phosphorylates Akt, leading to its activation. Activated Akt then phosphorylates and activates mTOR (mechanistic target of rapamycin). mTOR exists in two complexes: mTOR complex 1 (mTORC1) and mTOR complex 2 (mTORC2). In this context, mTORC1 is particularly interesting because it phosphorylates p70S6K, a kinase that regulates various cellular functions.

**Figure 2:**
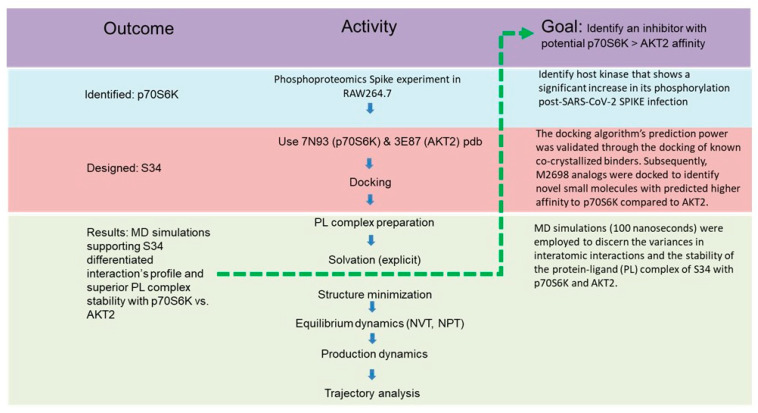
The multistep workflow performed in this publication. This study began with phosphoproteomic analysis (blue) of 1318 proteins post-SPIKE transfection, highlighting p70S6K for further investigation. Using its co-crystallized structure with M2698 from the RCSB PDB, molecular docking and MD simulations were conducted (red) following by the molecular dynamic simulation (green) using GROMACS tutorial (http://www.mdtutorials.com/gmx/complex/index.html).

Our study integrates multiple disciplines to identify a novel kinase targeted by a known inhibitor as a potential anti-COVID target (Figure 2). We initiated our investigation by examining the phosphorylation levels of p70S6K following SPIKE transfection of RAW264.7 macrophage cells. The underlying hypothesis posited that an elevation in phosphorylation at four residues situated on the C-terminal autoinhibitory domain would induce conformational changes facilitating the activation of p70S6K [27]. Additionally, phase I clinical trials involving patients with advanced cancer revealed that the inhibition of p70S6K was well tolerated [28]. The choice of p70S6K was influenced by the availability of a solved crystal bound to a potent ligand, M2698. M2698 is an oral dual Akt/p70S6K inhibitor with an IC50 of 1.1 nM for both targets. However, it was found to cause psychosis side effects in phase I clinical trials, possibly due to its impact on Akt2 [29]. We employed In silico calculations to study the interactions between M2698 and p70S6K as well as G95 (an Akt2 inhibitor with IC50 ranging from 6 to 190 nM) and Akt2. Our goal was to understand these interactions and then design an analog of M2698 predicted to exhibit enhanced binding affinity specifically to p70S6K. The in silico calculations entailed docking of a small library of M2698 analogs and G95. Subsequently, molecular dynamics (MD) simulations were conducted to assess the stability of the protein–ligand (PL) complex.

## 2. Material and Methods

### 2.1. Host Kinase Phosphorylation Panel Assay

The RAW264.7 macrophage cell line was treated with SARS-CoV-2 spike protein (SINO Ltd., Beijing China) for four time points: 0, 15, 30, and 60 min. Cell lysates were applied to the Phospho Explorer Antibody Array and date analysis (Full Moon BioSystems, Sunnyvale, CA, USA). The Phospho Explorer Antibody Array was used to explore the changes in 1318 proteins related to multiple signaling pathways and biological processes, and a ratio computation was used to measure the extent of protein phosphorylation: phosphorylation ratio = phosphorylated value/unphosphorylated value.

### 2.2. STRING Analysis

STRING analysis (https://string-db.org, access: 7 July 2023) generates a network diagram that visualizes the anticipated interactions among a list of user-specified proteins. In this representation, proteins are depicted as network nodes, while the connections between them signify functional associations [30]. To initiate the analysis, an 11-protein list was compiled encompassing two proteins from the PI3K/mTOR pathway (mTOR-S6K) and nine proteins recognized for their connection to COVID-19, originated from the DISEASES database (https://diseases.jensenlab.org/, access: 1 June 2023). This list was used as input for STRING analysis, which obtained protein-protein interaction (PPI) information through various prediction methods, including experimental data and co-expression techniques. The resultant networks were constructed with a confidence cutoff of 0.4, determining the threshold for an interactor to be considered authentic and thus included in the network.

### 2.3. Missing Loops Prediction Using SWISS-Model

SWISS-MODEL (http://swissmodel.expasy.org, access: 11 February 2023) serves as a platform for the automated comparative modeling of three-dimensional (3D) protein structures. To address missing protein loops in p70S6K crystal structure bound to co-crystallization binder M2698 (a compound that advanced to clinical trials), we utilized SWISS-MODELand incorporated input data from AlphaFold DB [31].

### 2.4. MOE Docking

To assess the predictive capability of our in silico model, we utilized the Molecular Operating Environment software (MOE.2022.02, CCG, Montreal, QC, Canada). This involved validating the model by comparing the pose of the co-crystallized M2698 against a small library of M2698 conformations (generated by MOE) docked into the p70S6K structure, utilizing default parameters. The SWISS-MODEL of the p70S6K structure underwent preparation for docking using MOE/Quickprep with all default values. A small library consisting of twenty-five M2698 analogs and their respective conformations, generated using MOE, were then subjected to docking.

After confirming the predictive power of the in silico model, a subsequent docking experiment was conducted involving M2698 and its close analogs. Additionally, G95, an Akt2 co-crystallized ligand (PDB:3E87), was docked in p70S6K (PDB:7N93) to gain further insights into the disparities between Akt2 and p70S6K binding pockets. The calculated docking score, which considers hydrophobic, steric, and electrostatic interactions, provided an estimate of the binding strength, serving as a proxy for the ligand binding free energy. During the docking process, we utilized the MOE template docking that aligns the conformation of M2698 analogs to a substructure of M2698 (specifically, M2698 without the carboxamide). An additional docking procedure was conducted in Akt2 (PDB:3E87) employing G95 for substructure alignment. This approach aimed to enhance our understanding of the distinctions in the binding pockets between the two proteins.

### 2.5. OCA

OCA serves as a database for protein structure and function (http://oca.weizmann.ac.il, access: 10 December 2023). The focus of OCA analysis centers on ligand–protein contacts with the aim of predicting the stability of protein–ligand complexes. In our study, we utilized this tool to gain insights into the impact of modifying specific atoms on the ligand, discerning how such modifications can either stabilize or destabilize the protein–ligand complex [32].

### 2.6. GROMACS

GROMACS, an open-source software designed for molecular dynamics (MD) simulations, is employed for studying the dynamics of proteins, whether in isolation or in the presence of small molecules [33]. Our MD simulations utilized GROMACS version 2021.4-Ubuntu-2021.4-2 and the Charmm36 force field. Hydrogen atoms were added to the X-ray structure using Avogadro [34]. CGenFF [35] was used to assign parameters and charges for ligand atoms, while pdb2gmx generated coordinates for the protein. The protein–ligand complexes were immersed in an octahedron box and solvated using the tip3p water model [36]. Solvent molecules were substituted with counterions until the system achieved neutrality. Subsequently, the solvated systems underwent the steepest descent minimization of 50,000 steps. Equilibration occurred in two stages, with the first involving NVT ensemble equilibration for 550,000 steps, followed by NPT ensemble equilibration for an additional 550,000 steps. The production run was executed with 2 fs time steps and 50,000,000 steps, totaling 100 ns of simulation time, with coordinates saved every 10.0 ps for analysis. The system size for the p70S6K structure (PDB:7N93) simulation comprised 45,600 atoms, while the system size for the Akt2 structure (PDB:3E87) simulation comprised 46,981 atoms.

The outcomes of MD simulations comprised trajectories or snapshots of the protein–ligand complex employing the Charmm36 force field. This force field serves as a mathematical framework that describes the protein’s energy based on its atomic coordinates [37]. Following the simulations, various analyses were carried out using the trajectory files obtained during simulation, including RMSD (root mean square deviation) and hydrogen bond interaction analysis. These analyses offered valuable insights into the differential stability and dynamics of the system upon ligand binding. The trajectory graphs were generated using the XMGRACE(Grace-5.1.25) software [38], and visualization was conducted using the open-source PyMOL molecular visualizer (Christoph Gohlke of the Laboratory for Fluorescence Dynamics, University of California, Irvine).

Binding affinities of S34 to both targets were estimated using the snapshots from molecular dynamics simulations collected previously. The first half of the trajectory was discarded, and binding enthalpies were estimated for each frame collected between 50 and 100 ns. The affinity estimates were calculated using the MMPBSA approach. GMX MMPBSA utility allows necessary file format conversion of the GROMACS trajectory and topologies to feed to MMPBSA calculation [39]. This utility was used to compute the end-state free energy calculations using MMPBSA.py from AmberTools23 under the hood. For preparing the systems to run end-state free energy calculations, the trajectories were first centered and periodic boundary conditions were removed, followed by the removal of water and ions. A similar approach was used to process the topology files before running the end-state energy estimation.

## 3. Results

### 3.1. Spike Experiment

To investigate the impact of SARS-CoV-2 on cell signaling-related proteins, a global phosphoproteomics experiment was conducted in the RAW264.7 macrophage cell line. Biological triplicates of cells were harvested at four different time points post-SARS-CoV-2 spike transfection (0, 15, 30, and 60 min). Using a phosphoarray, each sample was partitioned and assessed for changes in global protein abundance or phosphorylation at various sites. Phosphorylation fold changes were determined by calculating the ratio of phosphorylation at various time points (15, 30, and 60 min) to the phosphorylation level at the 0 min time point, which served as a control for all subsequent comparisons.

The results of phosphorylation sites are summarized in Table 1, revealing significant alterations in specific sites on p70S6K. Notably, Phospho-Thr229 exhibited downregulation at 60 min, Phospho-Thr421 was downregulated at 15 min, and Phospho-Ser418 showed a substantial upregulation (3.48× fold increase) at 60 min post-spike transfection. These phosphorylation changes align with existing literature indicating that the activation of p70S6K involves phosphorylation at Ser411, Ser418, Thr421, and Ser424, located in the pseudo-substrate region/C-terminal lobe/autoinhibitory domain of the protein [40]. Moreover, in the inactive conformation, this domain serves an autoinhibitory function by folding over the N-terminal lobe [41]. These phosphorylation events, along with Thr389 (not tested in our assay), induce conformational changes disrupting the interaction between the C and N termini, exposing the activation loop and permitting phosphorylation of Thr299 and Thr389 [42]. Additionally, mitogen-induced phosphorylation relieves autoinhibition and induces a conformational change in p70S6K [43], facilitating its phosphorylation at Thr389 by mTORC1 and phosphorylation of Thr229 by phosphoinositide-dependent kinase 1 (PDK1), ultimately leading to full activation of p70S6K [44]. Finally, Pearson et al. suggest that phosphorylation of Thr229 in the catalytic domain and Thr389 outside of the catalytic domain are possibly the most critical sites for p70S6K kinase function [45]. A possible explanation for the reduction in Thr229 phosphorylation at 60 min may have to do with the prerequisite phosphorylation of Thr389 that exposes Thr229 for phosphorylation. Our experiment did not track Thr389 phosphorylation, so we cannot comment on the difference in its phosphorylation over time.

### 3.2. STRING Database Analysis

A network analysis of the Middle East Respiratory Syndrome Coronavirus (MERS-CoV) infection highlighted the upregulation of members in the PI3K/Akt/mTOR and Ras/Raf/MEK/ERK signaling pathways. Given the close resemblance of SARS-CoV-2 to the pathogenesis, genetic makeup, and clinical features of MERS-CoV [47,48], we sought to explore this further. Inspired by this network analysis, we queried the STRING database by including p70S6K (gene name RPS6KB1) and mTOR as representative proteins for the PI3K/mTOR pathway, along with a list of closely related COVID-19 proteins based on the DISEASES database [49].

**Figure 3:**
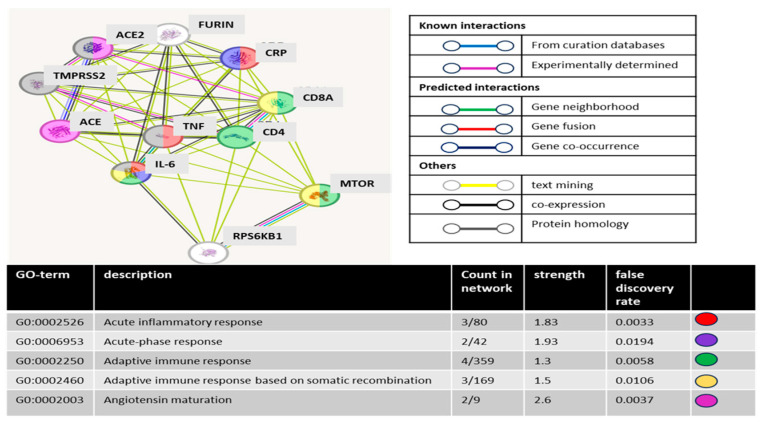
STRING pathway network analysis. Network visualization analysis screenshot shows proteins identified by our work mTOR-p70S6K and a set of proteins known to be associated with COVID-19 using DISEASES database. Network color nodes represent proteins, and network edges represent protein–protein associations and the method used for identifying the mentioned association.

The resulting network (Figure 3) revealed direct associations of RPS6KB1 with the cytokine IL6 and inflammatory response TNF [50], as well as glycoproteins involved in the T-cell receptor complex (CD4 and CD8a). Interestingly, the network indicated that many proteins were associated with adaptive immune (4/11, green) and acute inflammatory (3/11, red) responses. Significantly, the absence of pre-existing knowledge connecting RPS6KB1 to proteins associated with SARS-CoV-2, including ACE/ACE2/TMPRSS2, emphasizes the potential novelty and significance of our study.

### 3.3. In Silico Molecular Docking

We then chose to use in silico calculations to assess the binding affinity of M2698 for p70S6K in comparison to Akt2. These calculations involved molecular docking utilizing existing molecular structures and M2698. Our ultimate objective was to gain insights into the mentioned off-target toxicity of M2698, particularly involving Akt2, as observed in clinical settings. In pursuit of this, we investigated the interactions between M2698 and p70S6K versus M2698 and Akt2. This information laid the groundwork for the identification of M2698 analogs, such as S34.

### 3.4. Validation of the Docking Algorithm

The crystal structure of p70S6K (PDB:7N93) exhibited a few gaps in regions containing loops, which were subsequently filled using the proposed model from SWISS-MODEL (Figures S1 and S2). The final p70S6K structure, with completed loops, served as the basis for all in silico analyses.

**Figure 4:**
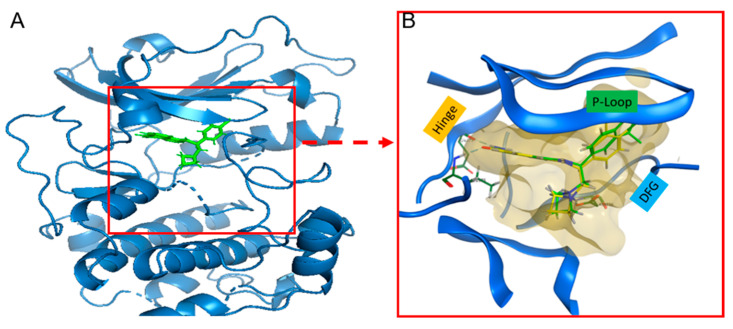
Best docked pose of M2698 with a zoom-in view into the crystallized binding pocket. M2698 conformers library was docked into the binding site of p70S6K cocrystalized with M2698 structure (PDB: 7N93). (**A**) Lowest RMSD of M2698 (green) docked in the p70S6K finalized structure. The best docked pose has a docking score S = −9.7 (the more negative, the more favorable) and RMSD = 1.03 Å compared to the co-crystallized ligand. (**B**) Zoom-in of the p70S6K binding site (beige) with the best docked M2698 pose (green) superimposed on co-crystallized M2698 (yellow). Known kinase motifs known to impact kinase activity are highlighted: Asp-Phe-Gly (DFG, cyan), hinge (gold), and p-loop [51].

The initial in silico task involved MOE docking (version 2022.02) to assess the predictive accuracy of the algorithm. A library of M2698 conformations generated by the MOE conformational search tool was docked into the p70S6K binding pocket. The best docked pose (Figure 4A, green) exhibited an RMSD of 1.03 Å from the co-crystallized pose (Figure 4B, yellow). Similar calculations were run for the Akt2 structure (PDB:3E87) and its co-crystallized ligand G95. Here too, the docked pose exhibited an RMSD of 0.54 Å compared to the co-crystallized pose. These results strongly support the continued application of the docking algorithm for investigating M2698 analogs.

### 3.5. OCA Recommendations for M2698 Modifications

Overcoming the challenges in kinase-targeted therapy, especially achieving compound selectivity, is crucial. Clinical observations reveal that inhibition of the PI3K/Akt/mTOR pathway often leads to poor tolerability and a narrow therapeutic window. This is particularly evident in some pan-Akt and PI3K inhibitors, where the lack of tolerability may be linked to off-target inhibition of Akt2. A 2022 review reported that individuals undergoing phase 1 clinical trials with M2698 as monotherapy experienced anxiety, abnormal dreams, and, in one case, psychosis [28]. To mitigate M2698’s off-target binding to Akt2, we utilized the Overlapping Clustering Application (OCA) tool for ideating chemical modifications to M2698, intending to demonstrate superior binding to p70S6K compared to Akt2.

**Figure 5:**
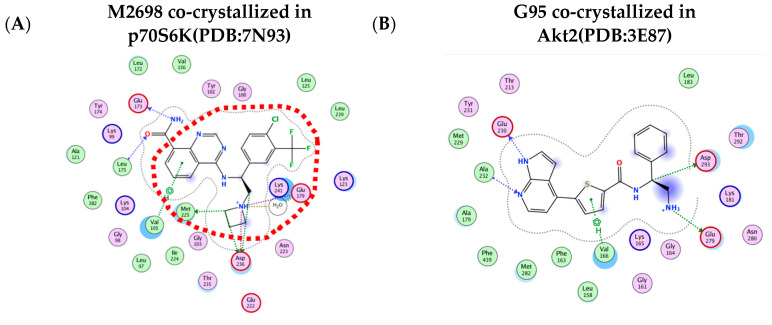

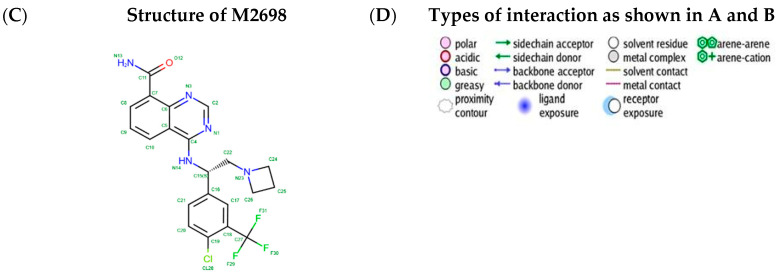
Known co-crystallized compounds and their interactions map. (**A**) Key interactions observed with the M2698 and p70S6K (PDB:7N93) binding sites: (1) the carboxamide moiety has a bidentate interaction with the Hinge region residues L175 and E173, while the second NH of the carboxamide forms an intramolecular hydrogen bond with the quinazoline nitrogen; (2) the substituted aromatic ring provides good mimicry with the nucleotide base of ATP and a pi-stacking interaction with V105; (3) the azetidine group fits into the shallow acidic pocket formed by E179, M225, and E222 and enhances selective kinase binding; and (4) the azetidine improves potency via a charged interaction with D236. The orange circle represents the common structure used for the M2698 SAR analogs. (**B**) Key interactions observed with the G95 and Akt2 (PDB:3E87) binding sites: (1) pyrrolopyridine has a bidentate interaction with hinge region residues E230 and A232; (2) the thiophene has pi-stacking with V166l; (3) a bidentate interaction between the amine and E279; and (4) sidechain hydrogen bond interaction with slightly charged carbon leading to the benzene ring on the back pocket. (**C**) Structure of M2698 with atom numbers. Atom colors: red—oxygen, blue—nitrogen, and green—fluorine atom. (**D**) A legend showing different types of interactions observed in (**A**,**B**).

The OCA tool [32] provided a detailed analysis of interatomic contacts and interface complementarity between M2698 and p70S6K. It also predicted the protein–ligand (PL) complex, giving it a stability score that can help assess the impact of chemical modifications to the ligand on protein binding. The results from the OCA analysis recommended modifying atom N13 (Table 2) located in proximity to E173 and E175 hinge residues (Figure 5C).

Specifically, replacing N13 with an aromatic/neutral atom is predicted to enhance protein–ligand complex stability with p70S6K. This recommendation was somewhat unexpected due to the existing salt bridge between M2698-N13 and E173-backbone-carbonyl and the intramolecular hydrogen bond of M2698-N13 with M2698-nitrogen-quinazoline. A possible explanation may have to do with the small hydrophobic patch (A121, V156, and L172), which unfavorably resides next to the polar carbonyl of the carboxamide in p70S6K (Figure 5A). Additionally, the concept of modifying N13 (Figure 5C) to eliminate one of the ligand’s interactions with the hinge residues (Figure 5B,C) was favored.

**Figure 6:**
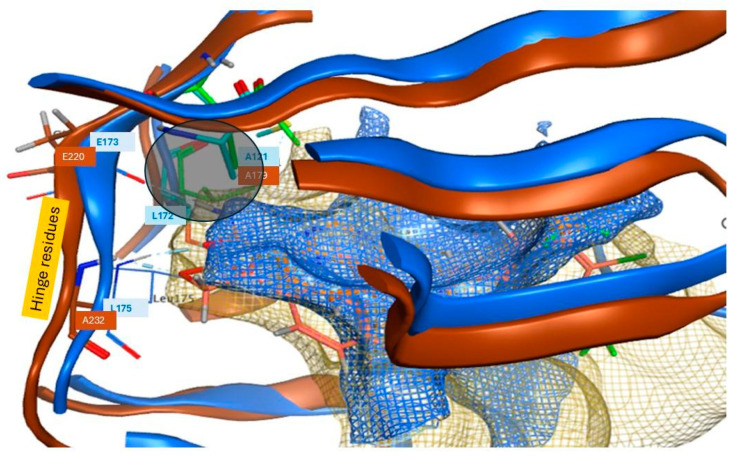
S34 predicted pose in p70S6K (blue) and Akt2 (brown). The p70S6K binding site is highlighted in blue mesh, with all residues labeled in blue. Highlighted p70S6K hinge residues E173 and L175 and hydrophobic residues A121 and L172 (located in a grey circle), representing the hydrophobic patch. In comparison, the Akt2 binding site is shown in beige, with hinge residues E220 and A232 labeled in brown, while the hydrophobic patch of Akt2 is marked in green.

**Figure 7:**
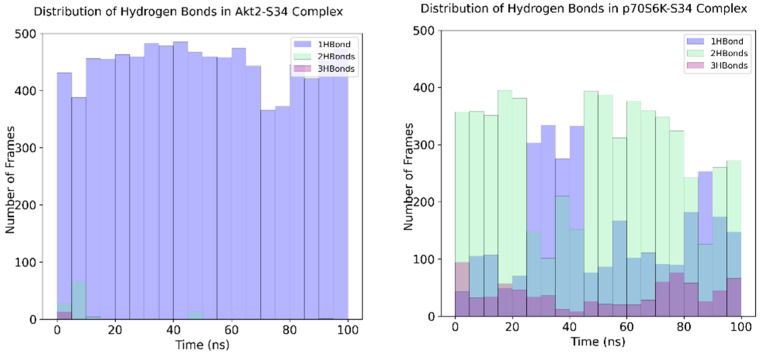
Distribution of the number of hydrogen bond interactions between protein and ligand during MD simulations of both complexes: Blue bar: number of frames with one hydrogen bond present; green bar: number of frames with two hydrogen bonds present; purple bar: number of frames with three hydrogen bonds present. Distributions for the Akt2-S34 complex are shown in the left histogram, and distributions for the p70S6K-S34 complex are shown in the right. The entire simulation time in ns is binned in the *x*-axis, and the number of frames is plotted along the *y*-axis. Each bin represents 5 ns of simulation time. The average number of hydrogen bond interactions between Akt2 and S34 observed was one, and the number of hydrogen bonds between p70S6K and S34 was two.

**Figure 8:**
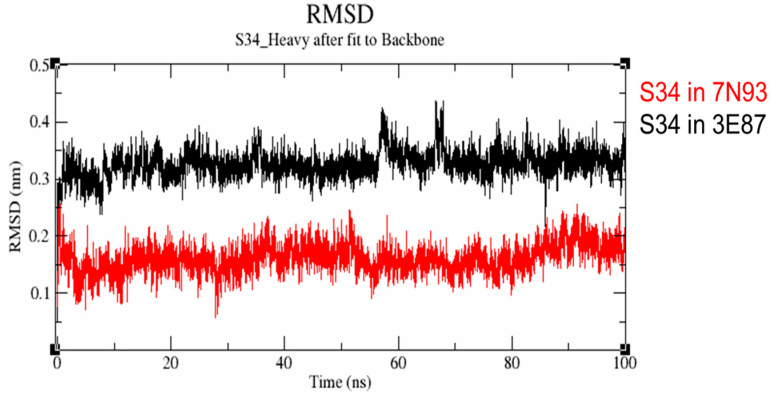
RMSD plot of protein backbone fluctuations after ligand binding. Black: RMSD plot of S34 binding to the Akt2 protein backbone; red: RMSD plot of S34 binding to the p70S6K protein backbone.

We postulated that the removal of one of the ligand’s interactions with the hinge residues might have a more pronounced impact on the M2698-Akt2 protein-ligand (PL) complex than the M2698-p70S6K complex. This is attributed to the relatively larger binding site of Akt2 (Figure 6), which can accommodate many more ligand conformations, potentially leading to a reduction in PL stability. The validity of this hypothesis was substantiated by the results of molecular dynamics (MD) simulations, showing more frequent formation of two or more hydrogen bonds throughout the simulation time (as measured by the number of trajectory frames containing two or more H-bonds) in the p70S6K-S34 complex compared to the Akt2-S34 system (Figure 7), along with a smaller ligand root mean-square deviation (RMSD) (Figure 8).

### 3.6. Molecular Docking

As previously mentioned, we used a small library of M2698 analogs (Figure S3) and a library of G95 conformations. Some M2698 analogs were derived from a 2021 publication [51], while others were newly designed by us. Although the OCA analysis suggested replacing N13 with aromatic or neutral atoms, we did not introduce aromatic modifications because we could not find an example of quinazoline bound to an aromatic group in the SureChEMBL database (https://www.surechembl.org/, access 23 December 2023). Docking into the binding sites of p70S6K and Akt2 was conducted using MOE template docking (MOE 2022.02). This approach ensured optimal alignment of the largest common fragment between the co-crystallized ligand and the docked molecule, enabling an assessment of the impact of N13 modifications on the predicted binding affinity. The rationale for designing the M2698 analog library for docking encompassed two key objectives: (1) mimicry of M2698-p70S6K interactions (depicted in Figure 5B) while introducing a neutral group at position N13 and (2) assessing the influence of incorporating a neutral group at position N13 on the docking scores in both p70S6K and Akt2. This modification could suggest reduced affinity, potentially mitigating off-target Akt2 toxicity [52].

The docking analysis of the co-crystallized ligands confirmed that our in silico models accurately ranked the co-crystallized compounds among the top three results based on docking scores for both p70S6K and Akt2. Furthermore, the RMSD values were notably low, measuring <1.07 Å for p70S6K and 0.58 Å for Akt2, indicating close alignment between predicted and experimental ligand binding poses. This outcome suggests the predictive nature of the in silico model.

The analog S34, featuring an oxetane moiety instead of a carboxamide group (Figure S3), exhibited a docking score of −9.4 when docked in p70S6K. This score is comparable to that of M2698 (−9.7), the co-crystallized ligand that ranked first in p70S6K. S34 exhibited a higher docking score (−7.78) compared to p70S6K (4) (−8.55), which ranked first in Akt2. Notably, M2698 was ranked seventh in the Akt2 docking list (information not included), possibly due to its limited structural overlap with the co-crystallized G95 structure that was used in the in silico template docking.

### 3.7. Molecular Dynamics (MD) Simulations

Next, we wanted to understand the changes in protein stability upon ligand binding using S34 complexes with p70S6K and Akt2 following 100 ns MD simulations. It was previously noted that ligand binding can induce changes in protein conformation, which can lead to an increase in protein stability [53]. Moreover, MD simulation can determine the dynamics of the PL complex at the atomistic level in a time-dependent manner [54]. This could be helpful in evaluating a ligand’s SAR (structure activity relationship) [54].

### 3.8. GROMACS Software for MD Simulations

We utilized GROMACS (version 2021.4) system trajectories for post-simulation analysis to assess the stability of the protein–ligand (PL) complex over the course of the simulation [55]. Subsequent analysis used methods that gauged PL stability and monitored the number of hydrogen bond interactions throughout the MD simulation. A higher count of hydrogen bond interactions could suggest stronger binding, potentially contributing to enhanced complex stability [56].

#### 3.8.1. Hydrogen Bond Interaction Analysis during MD Simulations

Hydrogen bond interactions are important biomolecular physical interactions in aqueous solutions [55]. Though context-dependent, hydrogen bond interactions contribute favorably to protein stability [57]. M2698 was noted to also have hydrogen bond interactions (Figure 5A) with E173 and L175 located on the p70S6K hinge. S34 is expected to have at least one less hydrogen bond interaction due to the carboxamide replacement with oxetane. During the MD simulations, we tracked the number of hydrogen bond interactions of S34 with residues located in the p70S6K (PDB:7N93) and Akt2 (PDB:3E87) binding sites. The analysis of hydrogen bonds (Figure 7) revealed that S34 forms an average of two hydrogen bond interactions with the pocket of p70S6K, whereas it only forms a single hydrogen bond interaction with Akt2. The reduction in the number of hydrogen bond interactions is in line with the RMSD values calculated at Figure 8.

#### 3.8.2. Root Means Square Deviation (RMSD) During MD Simulations

RMSD provides a quantitative metric that assesses alterations in the coordinates of protein backbone (BB) atoms between the initial and final conformational states throughout a simulation. Specifically, RMSD deviations observed during the simulation provide valuable insights into the stability of the protein. A higher RMSD deviation generally indicates a greater likelihood of protein instability [58]. Figure 8 shows variations in the protein backbone during ligand binding throughout the simulation.

In the context of the study, S34 demonstrated a stabilizing effect on p70S6K over the simulation period, exhibiting an RMSD of approximately 0.1 nm after 4 ns. It displayed minor RMSD fluctuations at the 28, 57, and 85 ns time points (Figure 8). The range of RMSD fluctuation was <0.25 nm, with backbone fluctuations ranging between 0.06 and 0.25 nm. Conversely, when S34 bound to Akt2 (Figure 8), the average RMSD was approximately 0.3 nm. Fluctuations in RMSD were more prominent at 60, 68, and 83 ns, which subsequently stabilized at ~0.32 nm. The range of RMSD fluctuation was <0.42 nm, with backbone fluctuations ranging between 0.24 and 0.42 nm. The fluctuations observed in RMSD can be ascribed to the disparate occurrences of hydrogen bonds during the interaction of S34 with two distinct proteins: p70S6K and Akt2. As noted above, S34 forms two hydrogen bonds on average when complexed with p70S6K (PDB:7N93), in contrast to only one hydrogen bond with Akt2 (PDB:3E87). The inference drawn from this could suggest that the S34-p70S6K complex may exhibit greater stability than the S34-Akt2 complex. This aligns well with the study’s overarching objective to underscore S34 binding differences in both proteins and justify the in silico work carried out in this study.

#### 3.8.3. Free Binding Energy Calculations Using MMPBSA

Free energy values for each system are provided in Table 3. Binding affinities were calculated using Equation (1) as follows:

(1) $$\Delta G_{b}\;=G_{complex}\;-G_{receptor}$$

It was observed that the total free energy (*G_complex_*) of the S34-p70S6K complex was lower than the total free energy of the S34-Akt2 complex. This was attributed to the lower free energy of the p70S6K as well as much more favorable ligand interactions in this complex. This difference resulted in almost 2-fold favorable binding affinity (Δ*G_b_*) of S34 in the p70S6K binding pocket compared to that in S34 in the Akt2 binding pocket. The difference in this binding affinity reflects the observation in the number of protein-ligand hydrogen bonding interactions at any instant in both the complexes, as shown in Figure 7.

### 3.9. Specificity Analysis

To increase the ligand affinity to p70S6K vs. Akt2, we hypothesized that reducing the number of hydrogen bond interactions between the ligand and residues located in the hinge region (from 2 to 1) would lead to the design of S34.

#### S34 Specificity Using MOE Docking

To assess the specificity of S34, we docked a library of S34 conformations into a selection of 156 kinases from MOE’s Kinase Protein Database, ensuring that they had less than 40% sequence homology among them as a way to increase kinase diversity. The top 10 best-scoring kinases, based on MOE’s docking score (S), are presented in Table 4. Notably, p70S6K was predicted to rank third, following G protein-coupled receptor kinase 2 (GRK2) and mitogen-activated protein kinase 3 (GLK), which aligns with our previous findings. Akt2 also demonstrated binding, ranking at position 99 (PDB:106K). Interestingly, GLK, p70S6K (S6K1), and protein kinase 1, which all ranked within the top 10, are involved in the mTOR signaling pathway. This suggests that S34 could target multiple proteins within the mTOR pathway, potentially offering a more robust inhibition of the pathway compared to targeting p70S6K alone.

An additional method used to assess S34 specificity involved the KLIFS software, version: 3.2) which enables the comparison of interaction patterns between kinase inhibitors and the key interactions that determine kinase inhibitor selectivity. The KLIFS results suggested that M2698 bound to three different kinases: p70S6K, PKACα, and Akt1 (Figure S4). Kinome mapping of these kinases revealed that all three belonged to the AGC sub-family (Figure S5). Interestingly, S34 did not show binding to any kinases, which may be attributed to the KLIFS’s ligand classification criteria. If S34 does not meet these specific criteria, it might not be represented in the database.

## 4. Spike Variants and Impact on p70S6K Phosphorylation

COVID-19 infection triggers the release of cytokines and inflammatory responses that activate signaling pathways common to diabetes, cancer, chronic kidney diseases (CKDs), and cardiovascular diseases (CVDs). Key pathways include MAPK1 (mitogen-activated protein kinase), EGFR (epidermal growth factor receptor), mTOR (mammalian target of rapamycin)/PI3K (phosphatidylinositol-3-kinase), and Fc epsilon receptor RI (FCERI). These pathways activate the innate immune system by promoting cell growth, division, survival, proliferation, cyclin synthesis, and angiogenesis. Cytokines such as IL-1β, IL-6, IFN-γ, and TNF-α are significantly elevated and could lead to a cytokine storm [59].

SARS-CoV-2 mutations are constantly evaluated, and numerous variants are classified as variants of concern (VOCs). VOCs have been attributed to greater disease transmission and virulence as well as a reduction in the efficiency of treatments and vaccinations. The Delta and Omicron variants, considered to be VOCs, have the highest number of mutations, particularly in the spike protein’s receptor-binding domain, which enhances the virus’s ability to bind to human ACE-2 receptors. Both the Delta and Omicron variants are highly effective in dysregulating signaling pathways, with Delta being particularly devastating, resulting in a higher number of infections and deaths. However, the precise molecular mechanisms by which SARS-CoV-2 variants impact signaling pathways remain an active area of scientific investigation [59].

## 5. Conclusions

COVID-19 persists as a highly transmissible infectious disease, presenting an ongoing and substantial public health challenge. The emergence of new variants, characterized by previously unseen genetic mutations, introduces the potential for varied pathologies. The rapid proliferation of coronavirus cases has spurred the research community to actively seek potent molecules capable of inhibiting the virus’s activity. The imperative lies in identifying effective strategies to mitigate the impact of this contagious pathogen on global public health.

**Figure 9:**
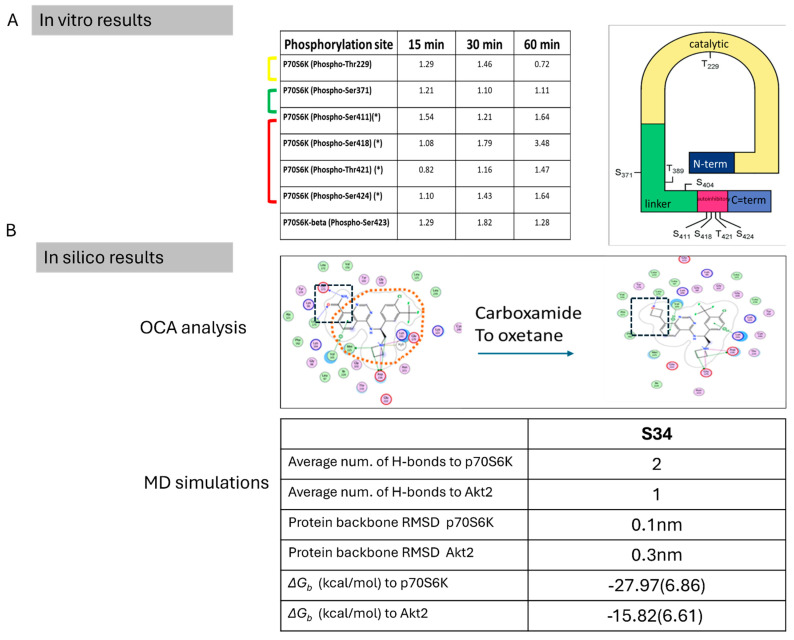
Results summary. This figure summarizes key results from both the in vitro phosphoarray experimental results (**A**) p70S6K has eight phosphorylation sites that regulate its activity of which six were tested here. All noted phosphorylation levels are compared to time 0 (baseline). (*) marks phosphosites that resides withing the autoinhibitory domain at the carboxylic terminus of the kinase. The yellow parenthesis represents residues located in the catalytic domain, green in the linker domain and red in the autoinhibitory domain of p70S6K. (**B**). The in silico results include OCA analysis, which highlighted N13 (black square) as the atom to be modified to improve protein-ligand stability. Orange circle represents the common structural part between M2698 and S34. Additionally, results from 100 nsec molecular dynamics (MD) simulations are summarized, presenting the average number of hydrogen bond interactions observed, RMSD (Root Mean Square Deviation), and free binding energy calculated using MMPBSA (Molecular Mechanics Poisson-Boltzmann Surface Area).

Our research involved a synergistic approach (Figure 9) [43,46,60], integrating wet bench experiments with in silico structural-based work to identify a novel host protein kinase and a specific inhibitor as a potential anti-COVID-19 target. In our wet bench experiment, we performed a comprehensive phosphoproteomics analysis utilizing RAW264.7 macrophage cells at four specific time points (0, 15, 30, and 60 min) after spike infection, which revealed alteration in phosphorylation dynamics. Noteworthy findings include the downregulation of Phospho-Thr229 and Phospho-Thr421, along with a substantial upregulation (3.48× folds) of Phospho-Ser418 post-SPIKE transfection. These observed changes in phosphorylation levels align with established knowledge regarding p70S6K activation, which necessitates phosphorylation at Ser411, Ser418, Thr421, and Ser424, as previously reported [40]. This correlation supports our findings and underscores the potential relevance of the identified phosphorylation events in the context of COVID-19.

The S6K protein family comprises S6K1 and S6K2, with S6K1 exhibiting two isoforms, namely, p70S6K and p85S6K1, generated through alternative translation initiation sites. Notably, these proteins play a pivotal role in modulating the mTOR pathway. The mTOR/S6K signaling cascade is implicated in various pathological conditions, including diabetes, cancer, and obesity, as it actively stimulates protein synthesis and cell growth [61]. Our study integrates p70S6K into the intricate network of mTOR/S6K signaling, underscoring its relevance and potentially offering new insights and therapeutic strategies for managing COVID-19. This notion is supported by recent research showing photothermal activity associated with reactive oxygen species (ROS) [62] is correlated with the downregulation of the Akt-mTOR-p70S6K pathway, which is known to regulate autophagy [63]. Although laser therapy for coronavirus treatment is still in its early stages, it presents an opportunity to selectively stimulate cellular processes involved in immune response regulation, such as autophagy.

The quest to identify active molecules represents a resource-intensive and time-consuming endeavor, leading to an increasing reliance on computational tools during the exploratory phase. In our study, we opted to leverage the crystal structures of p70S6K (PDB:7N93) in a complex with MSC2363318A-1 and the crystal structures of the kinase domain of Akt2 in a complex with G95 (PDB:3E87). The p70S6k-co-crystallized ligand, also called M2698, is a dual Akt/p70S6K compound with an IC50 of 1.1 nM for p70S6K and Akt2. M2698 has garnered attention as a clinical candidate in anti-tumor clinical trials [28]. Our in silico work commenced with the validation of the docking algorithm. The main aim was to evaluate the algorithm’s ability to accurately predict the co-crystallized ligand’s pose. Remarkably, the docking outcomes consistently positioned the docked co-crystallized compounds at the highest ranks, showing a low RMSD compared to the original co-crystallized ligand. These results offer additional support for the continued utilization of the docking algorithm in assessing the binding affinity of M2698 analogs. The objective is to identify an analog with a higher predicted affinity towards p70S6K.

The OCA analysis provided valuable insights into potential chemical modifications of M2698 aimed at ideating compounds that show increased affinity to p70S6K. The analysis recommended modifications to atom N13 (Figure 5C), strategically located near residues on the hinge region. In response to these recommendations, a focused library of M2698 analogs was designed, with particular attention given to S34. The underlying hypothesis was that S34 is likely to show reduced affinity to Akt2 following the elimination of one interaction with residues located in the hinge region. This conjecture is based on Akt2’s relatively larger binding site and the fewer hydrogen bonds observed with its co-crystallized ligand, G95, in PDB:3E87 (Figure 5B).

Subsequently, S34 was selected for MD simulations to gain valuable insights into its dynamics and interactions within the binding pockets of both p70S6K and Akt2. Specifically, we tracked the number of hydrogen bond interactions to elucidate the structure–activity relationship (SAR), examined the RMSD of the ligand at the beginning and end of the simulation, and assessed the impact on protein–ligand (PL) complex stability. The results, depicted in Figure 7, unveiled distinct hydrogen bond interaction patterns. Notably, when bound to p70S6K, S34 exhibited an average of two hydrogen bond interactions, while only one hydrogen bond interaction on average was observed when bound to Akt2. Additionally, the simulations monitored the RMSD values throughout the trajectories (Figure 8), providing a quantitative measure of changes in the backbone coordinates of the protein induced by ligand binding. The low RMSD values (~0.15 nM) of S34 in p70S6K compared to S34 in Akt2 (~0.33 nM) throughout the simulations underscore the heightened stability of the p70S6K-S34 PL complex. Lastly, MMPBSA calculations showed that the total energy of the S34-p70S6K complex was lower than the S34-Akt2 one. This was attributed to the lower total potential energy of the receptor as well as much more favorable ligand interactions (Table 3 and Figure 7). This difference resulted in almost 2-fold favorable binding affinity of S34 in the p70S6K binding pocket compared to that in the Akt2 binding pocket.

In conclusion, ongoing efforts are focused on developing inhibitors targeting components within the PI3K/Akt/mTOR pathway for therapeutic interventions across various diseases. However, many existing PI3K inhibitors exert a broad-spectrum effect, impacting all PI3K isoforms, while rapamycin, despite its inhibitory effects on the mTOR complex, exhibits promiscuity [64]. These observations underscore the importance of identifying selective inhibitors downstream of PI3K/mTOR proteins, such as p70S6K, to potentially mitigate the known cellular feedback loops or crosstalk between pathways that could influence therapeutic outcomes [65].

The primary objective of our work is not to assert that S34 is the next anti-COVID target. Instead, our focus is on demonstrating the effectiveness of this combined approach in discovering novel targets and potential repurposing opportunities, which could be relevant across various therapeutic areas, including but not limited to COVID-19. Our study identified p70S6K as a potential anti-COVID target. Furthermore, it highlighted a ligand (S34) with potentially superior efficacy towards p70S6K. This combined approach holds promise for expediting the identification of active ligands, serving as a valuable starting point for further exploration in developing anti-COVID therapeutics. However, while computational evaluations offer valuable insights and a potential foundation for anti-COVID drug discovery, translating these findings into effective therapeutics necessitates comprehensive validation through rigorous experimental studies. Therefore, integrating both computational and experimental approaches is essential for advancing potential targets against COVID-19 and its related CRS and long-term implications.

## Figures and Tables

**Table 1:** Phosphorylation level and different sites in p70S6K post-SPIKE transfection of RAW246.7 macrophage cells.

Phosphorylation Site	15 Min	30 Min	60 Min
p70S6K (Phospho-Thr229)	1.29	1.46	0.72
p70S6K (Phospho-Ser371)	1.21	1.10	1.11
p70S6K (Phospho-Ser411) (*)	1.54	1.21	1.64
p70S6K (Phospho-Ser418) (*)	1.08	1.79	3.48
p70S6K (Phospho-Thr421) (*)	0.82	1.16	1.47
p70S6K (Phospho-Ser424) (*)	1.10	1.43	1.64
p70S6K beta (Phospho-Ser423)	1.29	1.82	1.28

p70S6K has eight phosphorylation sites that regulate its activity [43,46], of which six were tested here. All noted phosphorylation levels are compared to time 0 (baseline). (*) marks phosphosites that reside within the autoinhibitory domain at the carboxyl terminus of the kinase.

**Table 2:** OCA normalized complementarity as a function of atomic substitution for M2698.

Ligand Atom	Atom Class	PL Complex Stability Score
**N13**	**Aromatic**	***0.93***
**N13**	**Neutral**	***0.93***
**N13**	**Aromatic-Neutral**	***0.93***
*N13*	*Hydrogen Bond Acceptor*	*0.8*

**Bold** labeled score indicates atomic substitution, which could stabilize the M2698-p70S6K complex. *Italic* labeled score indicates atomic substitution, which could destabilize the M2698-p70S6K complex.

**Table 3:** Free energy values obtained from the MMPBSA end-point calculation. All values are provided in kcal/mol. The uncertainties in the binding affinity estimates are provided within the parentheses.

System	*G_complex_*	*G_receptor_*	*G_ligand_*	Δ*G_b_*
S34-Akt2	515,682.7	515,728.44	−29.92	−15.82 (6.61)
S34-p70S6K	487,826.2	487,897.03	−42.85	−27.97 (6.86)

**Table 4:** Kinases predicted to bind S34 using in silico docking. The top 10 scoring kinases, their pdb, and their calculated S score are highlighted in the table.

Protein	PDB	S
GRK2	3KRW.A	−8.549
GLK	5J5T.A	−8.524
S6K1	3WE4.A	−8.469
Haspin	3E7V.A	−8.407
IRE1	4U6R.A	−8.321
Protein kinase 1	3MWU.A	−8.230
G Protein-coupled receptor kinase 6	3NYN.A	−8.200
TGF-beta receptor I	1PY5.A	−8.171
CDKL3	3ZDU.A	−8.155
DDR1	4CKR.A	−8.134

## Data Availability

All data is shared and open to the public in the manuscript.

## Supplementary Materials

The following supporting information can be downloaded at https://www.mdpi.com/article/10.3390/cells13211760/s1. Figure S1. Finalized model of 7N93; Figure S2. Modeled statistics; Figure S3. M2698 analogs used for docking in 7N93 crystal structure; Figure S4. Kinases predicted to bind M2698 or close analogs (Tanimoto > 0.75); Figure S5. Kinome view of M2698 predicted kinases binding.
