# Supplementary material for: p70S6K as a Potential Anti-COVID-19 Target: Insights from Wet Bench and In Silico Studies
Source: Cells. 2024 Oct 24;13(21):1760. doi: 10.3390/cells13211760 (PMC11545240; doi:10.3390/cells13211760)
Supplement: Supplementary file 1 [file cells-13-01760-s001.zip › cells-3191841-supplementary.pdf]

Supplement

7N93 missing loops introduced by SWISS-Model

The 7N93 crystal structure had few missing loops which were determined using SWISS model software and AlphaFold DB model of A0A485MZU6_LYNPA organism: Lynx pardinus (Iberian lynx) (Felis pardine) which had 99.71% sequency homology with 7N93. The GMQE (Global Model Quality Estimate), which indicates the level of accuracy of the model, was 0.91 which is high. Moreover, the Ramachandran plot, that looks at the statistical distribution of the backbone dihedral angles ϕ and ψ, suggested that all added loops were all within the legitimate range (figure 4).

<table>
<tr>
<td>
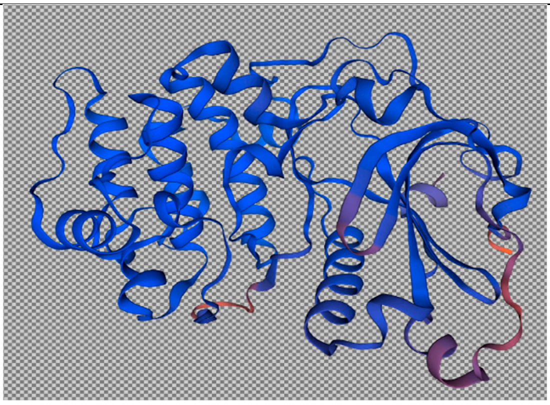

Figure S1. Finalized model of 7N93 where the blue parts are low disordered areas and red are high disordered, predicted areas
</td>
<td>
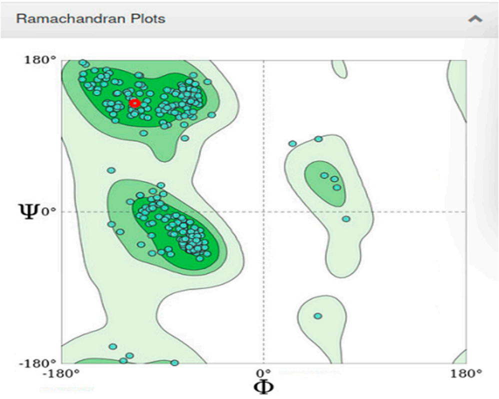

Figure S2. Modeled statistics. MolProbity score: 0.78 (MolProbity score is a weighted combination of the clash score, percent unfavored Ramachandran and bad sidechain rotamers.
Normal structure scores (-4) < score < 2.

Ramachandran Favored: 96.75% and Ramachandran Outliers: 0.95%. suggesting all amino acids possess an acceptable conformation.
</td>
</tr>
</table>

Figure S3
Small library of M2698 and analogs with their structure, docking score, best scoring pose docked in 7N93 (p70S6K) and observed interactions.

Molecule structure and name	Possess in 7N93 binding site	Interactions with residues in 7N93 binding site using MOE template module docking
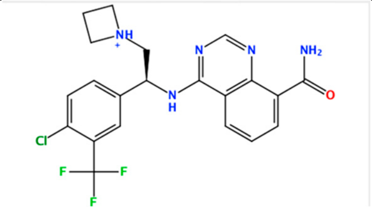 M2698 S=(-9.7)	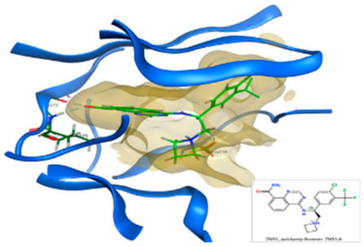	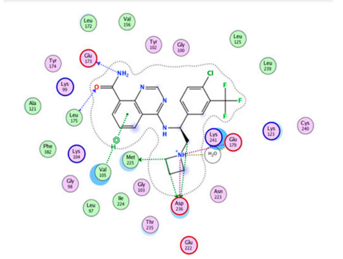
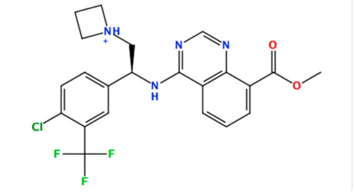 7N93_paper1 S=(-9.5)	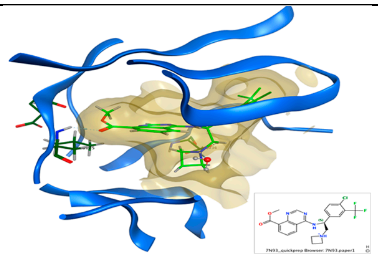	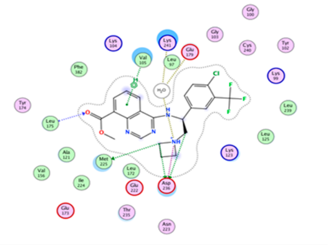
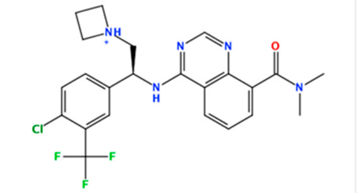 7N93_paper2 S=(-9.4)	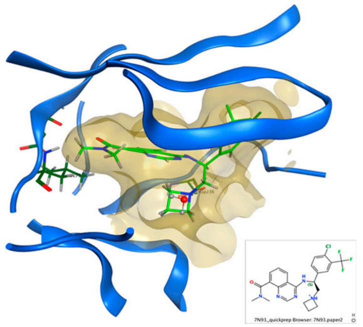	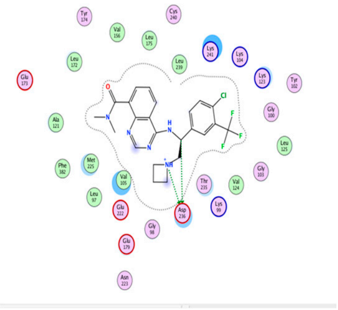
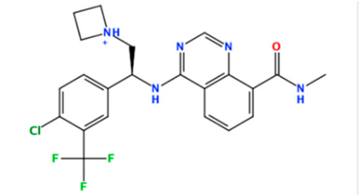 7N93_paper3 S=(-9.4)	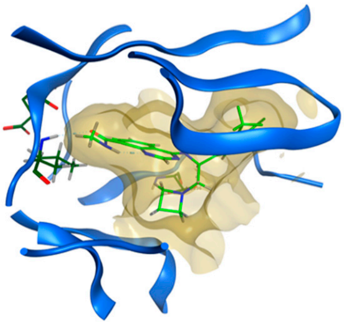	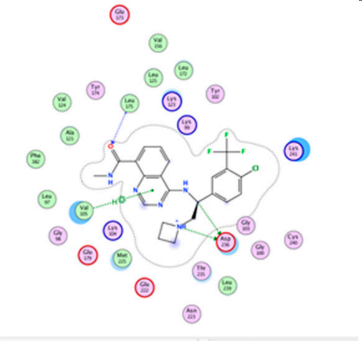

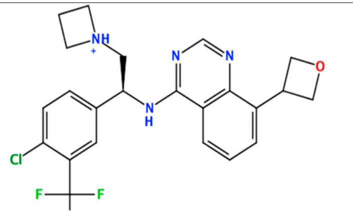<br>S34<br>S=(-9.4)	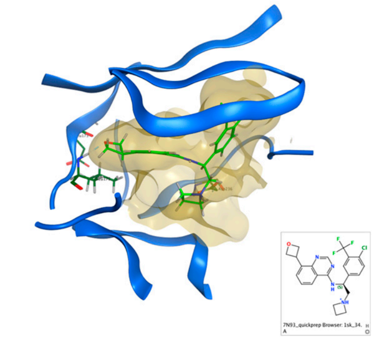	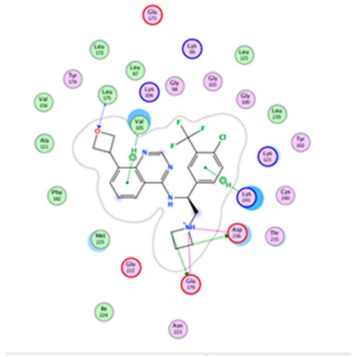
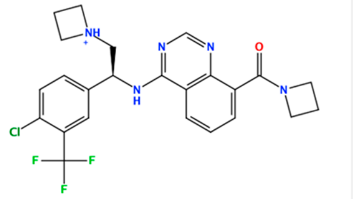<br>7N93(4)<br>S=(-8.3)	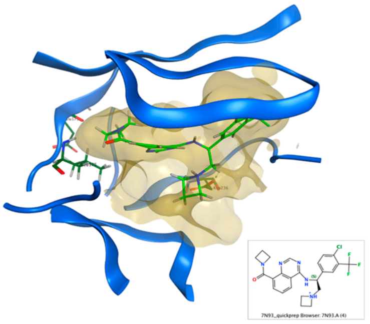	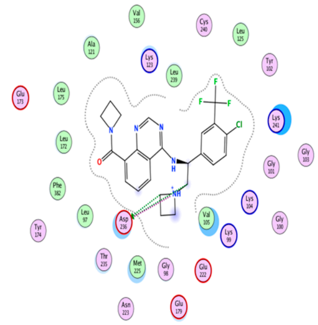

Figure S3. M2698 analogs used for docking in 7N93 crystal structure. From left to right: Compound name and docking score; Compound in binding site; 2D diagram of compound interactions with residues located in the binding pocket.

Figure S4, S5

KLIFS database

KLIFS database, containing 326 kinases, was used to determine kinase binding specificity. KLIFS is a kinase database that dissects experimental structures of catalytic kinase domains and the way kinase inhibitors interact with them. The KLIFS software enables the comparison of the interaction patterns of kinase-inhibitors and key interactions determining kinase-inhibitor selectivity. Using KLIFS analysis to predict kinase promiscuity, we observed that M2698 bound to three different kinases, including p70S6K and Akt1, as shown in Table 5. A kinome mapping of these kinases (p70S6K, Akt2, and PKACa) revealed that all three belong to the AGC sub-family (Table 7a). Interestingly, S34 did not show binding to any kinase proteins, which may be attributed to KLIFS's ligand classification criteria; if S34 does not meet these specific criteria, it might not be represented in the database.

NAME	FAMILY	GROUPS	PDB	CHAIN	SPECIES	LIGAND	TANIMOTO
PRKACA (PKACa)	PKA	AGC	4uja	A	Human	7-{(3S,4R)-4-[(5-bromothiophen-2-yl)carbonyl]pyrrolidin-3-yl}quinazolin-4(3H)-one	0.81
RPS6KB1 (p70S6K)	RSK	AGC	7n91	A	Human	4-{[(1S)-1-(3-fluorophenyl)-2-(methylamino)ethyl]amino}quinazoline-8-carboxamide	0.84
AKT1	Akt	AGC	3qkl	A	Human	N-{(2S)-3-[(3S)-8',9'-dihydro-1H,3'H-spiro[piperidine-3,7'-pyrano[3,2-e]indazol]-1-yl]-2-hydroxypropyl}-N-(2-ethoxyethyl)-2,6-dimethylbenzenesulfonamide	0.82
PRKACA (PKACa)	PKA	AGC	4uja	A	Human	7-{(3S,4R)-4-[(5-bromothiophen-2-yl)carbonyl]pyrrolidin-3-yl}quinazolin-4(3H)-one	0.81
PRKACA (PKACa)	PKA	AGC	4uj9	A	Human	7-[(3S,4R)-4-[4-(trifluoromethyl)phenyl]carbonylpyrrolidin-3-yl]-3H-quinazolin-4-one	0.78
RPS6KB1 (p70S6K)	RSK	AGC	7n93	A	Human	4-({(1S)-2-(azetidin-1-yl)-1-[4-chloro-3-(trifluoromethyl)phenyl]ethyl}amino)quinazoline-8-carboxamide	1

Figure S4. Kinases predicted to bind M2698 or close analogs (Tanimoto >0.75)

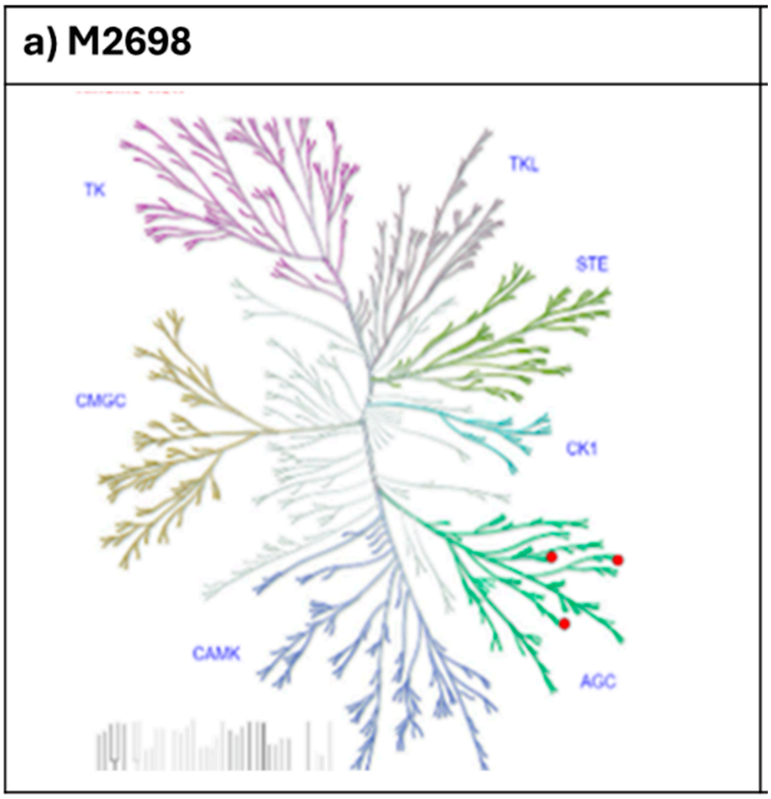

Figure S5. Kinome view of M2698 kinases binding: the branches lighted up represent kinases from the AGC family that binds M2698 or close analogs. The list of kinases that light up is displayed in Figure S4
